# Editorial: Hallmark of cancer: Resisting cell death

**DOI:** 10.3389/fonc.2022.1069947

**Published:** 2022-11-07

**Authors:** Yichao Zhu, Risheng Yang, Jacqueline H. Law, Muhammad Khan, Kenneth W. Yip, Qiang Sun

**Affiliations:** ^1^ Laboratory of Cell Engineering, Institute of Biotechnology, Beijing, China; ^2^ Research Unit of Cell Death Mechanism, Chinese Academy of Medical Science, Beijing, China; ^3^ Department of Medical Biophysics, University of Toronto, Toronto, ON, Canada; ^4^ Department of Zoology, University of the Punjab, Lahore, Pakistan; ^5^ Department of Cell and Systems Biology, University of Toronto, Toronto, ON, Canada

**Keywords:** cell death, ferroptosis, anoikis, apoptosis, entosis, cell-in-cell, hallmarks of cancer, drug resistance

In 2000, Douglas Hanahan and Robert Weinberg proposed 6 physiological features as the hallmarks of cancer (1), which evolved into 14 hallmarks with the rapid advances in cancer research over the past two decades (2). Among the long list of hallmarks, conceivably being further updated in the future, evading programmed cell death constitutes one of the founder mechanisms whereby tumors can establish successfully (1, 2). Whereas apoptosis was regarded as the primary form of programmed cell death two decades ago, it is currently well acknowledged that cell death can be executed through a plethora of programmed mechanisms. The latest recommendations by the Nomenclature Committee on Cell Death in 2018 proposed 13 distinct forms of programmed cell death, including classical forms of autonomous cell death, such as apoptosis, necroptosis, autosis, ferroptosis and netosis, and the emerging concept of non-cell-autonomous death, such as entosis, which is mediated by the formation of cell-in-cell structures (CICs) (3). Novel mechanisms of cell death, such as cuproptosis (4), are continually being identified. Given the pivotal role of resisting cell death in the development and progression of tumors, almost every form of cell death has been, more or less, implicated in human cancers. This Research Topic collected a set of research articles and comprehensive reviews related to the representative forms of cell death that are currently undergoing extensive investigation in multiple human cancers to promote the diagnostic and therapeutic applications of targeting cell death in the clinic.

## Ferroptosis

Research on ferroptosis, a novel form of programmed cell death driven by iron-dependent lipid peroxidation, has grown exponentially over the past years. Ferroptosis is regulated by multiple cellular pathways, including redox homeostasis, iron handling, mitochondrial activity, and energy metabolism (5). Jiang et al. explored the mechanisms underlying drug- and ferroptosis-resistance in advanced prostate cancer. They found that docetaxel-resistant prostate cancer cells developed tolerance to ferroptosis by TFAP2C-induced upregulation of lncRNA PCAT1, which subsequently activates the expression of SLC7A11, a Cys2/glutamate antiporter that functions as a negative regulator of ferroptosis (5). This works through the stabilization of c-Myc to promote SLC7A11 transcription and outcompetition of microRNA-25-3p, enhancing SLC7A11 translation by PCAT1. This research provides novel mechanistic insights into drug resistance in advanced prostate cancer, highlighting the therapeutic potential of ferroptosis in cancer treatment.

Although the induction of ferroptosis to limit tumor progression by directly inducing tumor cell death is promising, conservative voices also draw attention to the potential tumor promoting function of ferroptosis, which can create a pro-tumor microenvironment. Focusing on this issue, Bi et al. summarized the impacts of ferroptotic tumor cells on the tumor immune microenvironment. Thus far, ferroptosis inducers have been described to not only efficiently kill tumor cells, but also cause the death of anti-tumor immune cells, including CD8+ T cells, NK cells and DC cells, thereby evading anti-tumor immunity. Meanwhile, ferroptotic tumor cells can promote the infiltration and polarization of pro-tumor immune cells, including tumor-associated macrophages, regulatory T cells and myeloid-derived suppressor cells, which are generally resistant to ferroptosis. This analysis highlights the necessity to refine the specificity of ferroptotic therapy to target tumor cells and pro-tumor immune cells while sparing anti-tumor immune cells.

## Anoikis

Anoikis is a special form of apoptosis that is activated upon cell detachment and primarily mediated by the pro-apoptotic proteins Bid and Bim through the intrinsic apoptotic pathway. Alternatively, ECM detachment results in the release of mitochondrial Bit1 into the cytoplasm to trigger apoptosis (6) (Adeshakin et al). Zhu et al. review the factors contributing to anoikis resistance in glioma, such as adhesion molecules and signaling pathways including EGFR, IGFR, Trk, TGF-β, Hippo pathway, cytoplasmic proteins, the tumour microenvironment and protective autophagy. Therapy targeting these factors represents a potentially favorable strategy for the treatment of glioma.

## Cell-in-cell death

CICs, characterized by one or more cells inside another cell, are prevalent in many human cancers where tumor cells can internalize either other tumor or immune cells (7). CIC formation frequently leads to the death of the internalized cells (8) or the engulfers (9), and therefore was proposed as a novel type of programmed cell death (3) that could promote immune regulation (10, 11), tumor evolution and progression (12). Consistently, Wang et al. identified that the presence of CICs was an independent risk factor that was significantly associated with poor survival for patients with hepatocellular carcinoma, particularly in those with lower grades and at an early stage. This study further validated CIC-indexed functional pathology in the prognosis of cancer patients (13).

## Cancer immunotherapy and drug resistance

In recent years, the activation of cytotoxic immune effector cells (to kill tumor cells) using antibodies targeting immune checkpoints has proven to be an effective strategy for cancer therapy. The engagement of programmed cell death protein 1 (PD-1) with programmed death-ligand 1 (PD-L1) represents one of the best druggable immune checkpoints, which is frequently the target of oncogenic transformation evading anti-tumor immunity (14, 15). Wang et al. found that trastuzumab-treated gastric cancer patients expressing high ERBB2D16, a HER2 isoform without the 16^th^ exon, survived for a significantly shorter time than those with low ERBB2D16. This was associated with a strong immunosuppressive tumor microenvironment characterized by a high level of PD-L1/PD1 expression and impaired infiltration of CD3+ T cells. Yu et al. analyzed a cohort of patients with liver cancer treated with PD-1 inhibitor and tyrosine kinase inhibitors. They found that the treatment could cause immune-related adverse events manifested by impaired liver function, which was positively associated with increased C-reactive protein and IL-6 and decreased T and B subsets. Thus, protecting normal cells from cell death by immune therapy should also be taken into account while ensuring the effective killing of tumor cells.

## Conclusions

Although the papers included in this Research Topic reported the latest progress in several forms of cell death, they do not cover the full diversity of cell death processes. Other modes include necroptosis, autosis and netosis, which are being extensively investigated in cancers. This topic offers an inspiring note to encourage more valuable studies on the evasion of cell death in cancer. Of note, there are up to 14 hallmarks of cancer proposed, and in addition to this current Research Topic, Frontiers in Oncology has also sponsored topic collections covering another 9 hallmarks of cancer. We encourage readers to visit and explore.

## Author contributions

All authors listed have made a substantial, direct, and intellectual contribution to the work and approved it for publication.

## Acknowledgments

The editors thank all the authors and reviewers who contributed to the Research Topic and success of this special issue. QS was supported by grants from the National Key R&D Program of China (2022YFC3600100, 2019YFA0903801) and the National Natural Science Foundation of China (31970685).

## Conflict of interest

The authors declare that the research was conducted without any commercial or financial relationships construed as a potential conflict of interest.

## Publisher’s note

All claims expressed in this article are solely those of the authors and do not necessarily represent those of their affiliated organizations, or those of the publisher, the editors and the reviewers. Any product that may be evaluated in this article, or claim that may be made by its manufacturer, is not guaranteed or endorsed by the publisher.
